# Supplementary material for: Identifying and evaluating barriers for the implementation of machine learning in the intensive care unit
Source: Commun Med (Lond). 2022 Dec 21;2:162. doi: 10.1038/s43856-022-00225-1 (PMC9768782; doi:10.1038/s43856-022-00225-1)
Supplement: Supplementary file 1 — Reporting Summary [file 43856_2022_225_MOESM1_ESM.pdf]

nature research

Corresponding author(s): Ellie D'Hondt

Last updated by author(s): 14/10/2022

Reporting Summary

Nature Research wishes to improve the reproducibility of the work that we publish. This form provides structure for consistency and transparency in reporting. For further information on Nature Research policies, see our Editorial Policies and the Editorial Policy Checklist.

Statistics

For all statistical analyses, confirm that the following items are present in the figure legend, table legend, main text, or Methods section.

n/a	Confirmed	
☐	☒	The exact sample size (*n*) for each experimental group/condition, given as a discrete number and unit of measurement
☐	☒	A statement on whether measurements were taken from distinct samples or whether the same sample was measured repeatedly
☒	☐	The statistical test(s) used AND whether they are one- or two-sided<br>*Only common tests should be described solely by name; describe more complex techniques in the Methods section.*
☒	☐	A description of all covariates tested
☒	☐	A description of any assumptions or corrections, such as tests of normality and adjustment for multiple comparisons
☒	☐	A full description of the statistical parameters including central tendency (e.g. means) or other basic estimates (e.g. regression coefficient) AND variation (e.g. standard deviation) or associated estimates of uncertainty (e.g. confidence intervals)
☒	☐	For null hypothesis testing, the test statistic (e.g. *F*, *t*, *r*) with confidence intervals, effect sizes, degrees of freedom and *P* value noted<br>*Give P values as exact values whenever suitable.*
☒	☐	For Bayesian analysis, information on the choice of priors and Markov chain Monte Carlo settings
☒	☐	For hierarchical and complex designs, identification of the appropriate level for tests and full reporting of outcomes
☒	☐	Estimates of effect sizes (e.g. Cohen's *d*, Pearson's *r*), indicating how they were calculated

Our web collection on statistics for biologists contains articles on many of the points above.

Software and code

Policy information about availability of computer code

Data collection	n/a
Data analysis	The code for the AKI model and related experiments is published under the AGPL licence in a public github repository (http://dx.doi.org/10.5281/zenodo.7096747). The complete list of features used can also be retrieved there.<br><br>The code we used for the experiments on the Readmission model is available from Github (http://dx.doi.org/10.5281/zenodo.7096601) under an MIT license (which is the same type of license as the repository that it was forked from). The original repository was downloaded from the url https://github.com/apakbin94/ICU72hReadmissionMIMICIII.

For manuscripts utilizing custom algorithms or software that are central to the research but not yet described in published literature, software must be made available to editors and reviewers. We strongly encourage code deposition in a community repository (e.g. GitHub). See the Nature Research guidelines for submitting code & software for further information.

Data

Policy information about availability of data

All manuscripts must include a data availability statement. This statement should provide the following information, where applicable:

- Accession codes, unique identifiers, or web links for publicly available datasets
- A list of figures that have associated raw data
- A description of any restrictions on data availability

The questionnaire used for the VoC is repeated in Supplementary Note 1, and publicly available on github (http://dx.doi.org/10.5281/zenodo.7096767). The summarized questionnaire results are available in the article. Individual questionnaire results are not available because of confidentiality.

Data used in experiments were drawn from the Medical Information Mart for Intensive Care III (MIMIC-III, https://doi.org/10.13026/C2XW26). It is a single-center database containing 53423 distinct ICU stays for patients aged 16 and over between 2001 and 2012 at Beth Israel Deaconess Medical Center, in Boston, Massachusetts. It is available without cost to researchers who complete the appropriate training for data handling. Figures 1, 2 and 3 in the article have associated raw data which can be found in Supplementary Data 1, 2 and 3.

All other data are available from the corresponding author (or other sources, as applicable) on reasonable request.

Field-specific reporting

Please select the one below that is the best fit for your research. If you are not sure, read the appropriate sections before making your selection.

☐ Life sciences ☒ Behavioural & social sciences ☐ Ecological, evolutionary & environmental sciences

For a reference copy of the document with all sections, see nature.com/documents/nr-reporting-summary-flat.pdf

Behavioural & social sciences study design

All studies must disclose on these points even when the disclosure is negative.

Study description

Mixed methods. Qualitative voice-of-the-customer study on the potential of data analytics in healthcare (40 structured interviews). Complemented with a quantification of some of the issues raised through machine learning experiments on the MIMIC-III open dataset (loss of performance in ICU predictive models due to drift, failing to use extra inputs, scarceness of data, and deploying a model in a different context to the one it was built in).

Research sample

The VoC study was carried out with 17 hospitals and 7 MedTech companies in the period of May-December 2020. The focus was on the ICU environment, including units for neonatal (NICU) and pediatric (PICU) intensive care. ICUs involved were of various sizes: 5 had under 20 beds, 5 between 20 and 30, 2 between 30 and 40, and 3 had over 60 beds. For 2 further institutions, the interviewees were not connected to a particular ICU. The 7 MedTech companies provided insights into the landscape for IT support in hospitals and their ICUs. Four companies provide software solutions in ICUs, 1 is an ICU device manufacturer, 1 works on ICU and hospital-wide interoperability, and 1 provides remote monitoring for implantable cardiac devices. We spoke with a total of 40 interviewees in 29 structured interviews. With 3 stakeholders we carried out more than one interview (resp. 8, 4 and 1 additional interviews). Hospital staff generally filled in senior roles in their organisation (2 C-level, 4 heads of IT, 7 heads of ICU, 3 head nurses). We did not ask interviewee's ages. All had some clinical research activities, with 3 having the bulk of their workload in academic research. MedTech staff interviewees had varied roles: 1 project manager, 3 technical profiles, 1 CEO, 3 sales managers, 1 director market & offering, and 1 senior data scientist. Further details can be found in Tables 1-3.

Sampling strategy

Sampling prodedure was stratified and, within each stratum, convenience. We did not predetermine sample size, rather gathering as many interviews we could within the time available for their analysis. Data saturation was not considered formally.
There are indications of bias in our interview sample. When setting up interviews, we simply stated the topic to be data innovation in the ICU. There is some risk that those interviewed are self-selected as a sample with positive bias towards this topic. On the other hand, the VoC occurred in COVID-19 times causing extremely busy ICUs. It is unlikely that unresponsiveness was always due to disbelief or disinterest in health data innovation. Two hospitals were involved before the COVID-19 crisis hit in Europe. We asked them in May, respectively September 2020, how the situation impacted their views. The first responded that, aside from an acute and severe lack of resources, the potential of and issues with data analytics in ICUs remain much the same. The second gave insights in the novel patient monitoring needs of the typical COVID-19 patient, and the interplay with sepsis and delirium.

Data collection

The interviews were conducted by a data innovator and a former CEO from a MedTech company acting as a consultant, in the context of an innovation track at a large research centre in Belgium. One interview takes about 1-2h to conduct and its subsequent analysis about 1 day. Interview notes were taken on a laptop (blind typing), a mockup was shown on a laptop halfway through the interview. Interview analyses was carried out by typing out notes after the interview and structuring answers in an excel table.
The interview is structured according to the Double Diamond design process and consists of parts: introduction, current practices (team, workflow, infrastructure, data); unmet needs and wants (what is / is not working well, needs, future vision, role of data analytics); innovation pitch, attitude towards the proposed solutions, drivers and barriers (blocking factors for analytics and possible mitigations), closing. In this study innovations were introduced by way of mockups shown to the interviewees. By proposing the innovation only midway through the interview, we avoid biasing answers to the questions on the current state and needs towards the assumptions we want to test.

Timing

The work was executed in the midst of the COVID-19 pandemic. This had an impact on the availability of respondees and hence the timings of interviews. In Belgium, where most of the interviews took place, the peak of the first wave was early April and of the second wave early November. We did not contact anybody during these stressful periods. Instead, we focused on the quantitative part of the research (MIMIC-III experiments). Interviews occurred in two main batches: Late May - June (9) and September (12). One interview occurred in July, 2 in August, 3 in October and 2 in December. The interviews in October focused on nursing staff..

Data exclusions

No data was excluded in our VoC. For the MIMIC-III experiments we queried the database on the relevant datapoints and used those.

Non-participation

Of the 61 stakeholders we reached out to outside of these periods, 29 resulted in an interview, 23 were unresponsive, 6 were not available because of other priorities and 3 had to cancel interviews due to COVID-19 priorities.

Randomization

N/A

Reporting for specific materials, systems and methods

We require information from authors about some types of materials, experimental systems and methods used in many studies. Here, indicate whether each material, system or method listed is relevant to your study. If you are not sure if a list item applies to your research, read the appropriate section before selecting a response.

Materials & experimental systems

n/a	Involved in the study
☒	☐ Antibodies
☒	☐ Eukaryotic cell lines
☒	☐ Palaeontology and archaeology
☒	☐ Animals and other organisms
☒	☐ Human research participants
☒	☐ Clinical data
☒	☐ Dual use research of concern

Methods

n/a	Involved in the study
☒	☐ ChIP-seq
☒	☐ Flow cytometry
☒	☐ MRI-based neuroimaging
