# Supplementary material for: Identifying and evaluating barriers for the implementation of machine learning in the intensive care unit
Source: Commun Med (Lond). 2022 Dec 21;2:162. doi: 10.1038/s43856-022-00225-1 (PMC9768782; doi:10.1038/s43856-022-00225-1)
Supplement: Supplementary file 2 — Description of Additional Supplementary Files [file 43856_2022_225_MOESM2_ESM.pdf]

Description of Additional Supplementary Files

File Name: Supplementary Data 1

Description: Source data for Figure 1: AKI model performance as available data size varies, with and without extra features.

File Name: Supplementary Data 2

Description: Source data for Figure 2: AKI model performance as available data size varies, with and without TL.

File Name: Supplementary Data 3

Description: Source data for Figure 3: Readmission prediction model performance as available data size varies.
