# Supplementary material for: Identifying and evaluating barriers for the implementation of machine learning in the intensive care unit
Source: Commun Med (Lond). 2022 Dec 21;2:162. doi: 10.1038/s43856-022-00225-1 (PMC9768782; doi:10.1038/s43856-022-00225-1)
Supplement: Supplementary file 3 — Supplementary Information [file 43856_2022_225_MOESM3_ESM.pdf]

Identifying and evaluating barriers for the implementation of machine learning in the intensive care unit

Ellie D'Hondt[1, *], Thomas J. Ashby[1, *], Imen Chakroun[1, *], Thomas Koninckx[2], and Roel Wuyts[1, *]

[1]Exascience Life Lab, imec, Leuven, Belgium
[2]Independent consultant, Belgium
[*]Corresponding Authors: {tom.ashby, imen.chakroun, ellie.dhondt, roel.wuyts}@imec.be

Supplementary Note 1: VoC interview on ICU data innovation

Start of Interview

Stakeholder: …
Interviewee: …
Role: …
Date: …

INTRODUCTION (5')

Brief intros.

Context: eHealth innovation track. Supported by [X], a precompetitive research centre, a.o. supporting the transition of tech from research artefacts to market uptake.

Briefly explain the approach of the interview:

- Part 1: Understand current practices & needs.
- Intermezzo: Present our ideas on innovation in ICUs. . To avoid biasing your answers, we do this only after concluding Part 1.
- Part 2: . Gather your feedback on our ideas..

***PART 1**: FOR MEDICAL STAFF*

Current practices (15')

Q1: Which team do you work in, and what is your role in that team?

Q2: Please describe the typical patient population of the hospital you work in, and the clinical target group of your ICU.

Q3: What are the specifications of your ICU facilities? (Number of beds, layout of ICU, number of admissions per year, size of paramedical team)

Q4: Can you describe a typical day in your ICU ? How do you determine the daycare program for an average patient?

Q5: Which infrastructure is used to monitor patients?

PDMS system:
Central monitoring: yes/no
EMR system:

Q6: Which patient data is stored and in which way?

Q7: How do you use patient data in the day-to-day operation of your ICU?

Unmet needs/wants (10')

Q8: What works well today, which elements of your work are you satisfied with?

Q9: What could be better? What is missing in the current way of working?

NUDGE (only if no immediate answer, or to refine the answer given):

1. Where could you save time in the current workflow, and in what way? Where is there any loss of time now?
2. Where could you guarantee or improve safety or quality in the current workflow, and in what way?
3. Where could you improve upon clinical decision data in the current workflow, and in what way?

Q10: What do you understand by data analytics?

Q11: How do you use data and / or do data analytics tools support you in your day-to-day work and patient care?

NUDGE (only if no immediate answer, or to refine the answer given):
1. (Real-time) decision support
2. Personalized healthcare
3. Clinical studies
4. Anomaly detection

Q12: How do you see clinical decision making (and the IT that supports it) evolve over the next 5 years?

Q13: Why do you think data-driven solutions are only used to a limited extent in clinical care?

***PART 1**: FOR IT STAFF / MedTech*

Current practices (15')

Q1: Which team do you work in, and what is your role in that team?

Q2: Can you tell us about the IT team composition and way of working?

Q3: What are the specs of your IT hospital-wide infrastructure / products?

Q4: What about the IT infrastructure in / for the ICU?

PDMS system:

Central monitoring: yes/no

EMR system:

Q5: What role does patient data play in your hospital / in your products?

Q6: How is patient data made available within the hospital and outside of it(storage of data)?

PDMS ↔ EMR:
Time series frequencies:
Manual or automatic storage?
How long is data kept?

Unmet needs/wants (10')

Q8: What works well today, which elements of your work are you satisfied with?

Q9: What could be better? What is missing in the current way of working?

NUDGE (only if no immediate answer, or to refine the answer given):
1. Where could you save time in the current workflow, and in what way? Where is there any loss of time now?
2. Where could you guarantee or improve safety or quality in the current workflow, and in what way?
3. Where could you improve upon clinical decision data in the current workflow, and how?

Q10: What do you understand by data analytics?

Q11: How do you use data and / or do data analytics tools support you in your day-to-day work and patient care?

NUDGE (only if no immediate answer, or to refine the answer given):
1. (Real-time) decision support
2. Personalized healthcare
3. Clinical studies
4. Anomaly detection

Q12: How do you see the roll-out of hospitals and their IT systems evolving in the next 5 years?

Q13: Why do you think data-driven solutions are only used to a limited extent in clinical care?

Q14: How is *data analytics*[1] available to support hospital operations?

INTERMEZZO

Innovation pitch (5')

We believe there is a lot of potential in data-driven software solutions to improve IT-support for medical personnel. Despite innovative and groundbreaking research in this domain, we see little of this in day-to-day patient care. We would like to understand better why this is the case. Where are the pain points, and which of these pain points can be alleviated by better IT support? We have come up with a proposal for innovative software for data-driven ICUs which we present via mockup slides (cfr. below). We would like to solicit your feedback on this proposed innovation, as a way to concretise which pain points are resolved by it, and which remain.

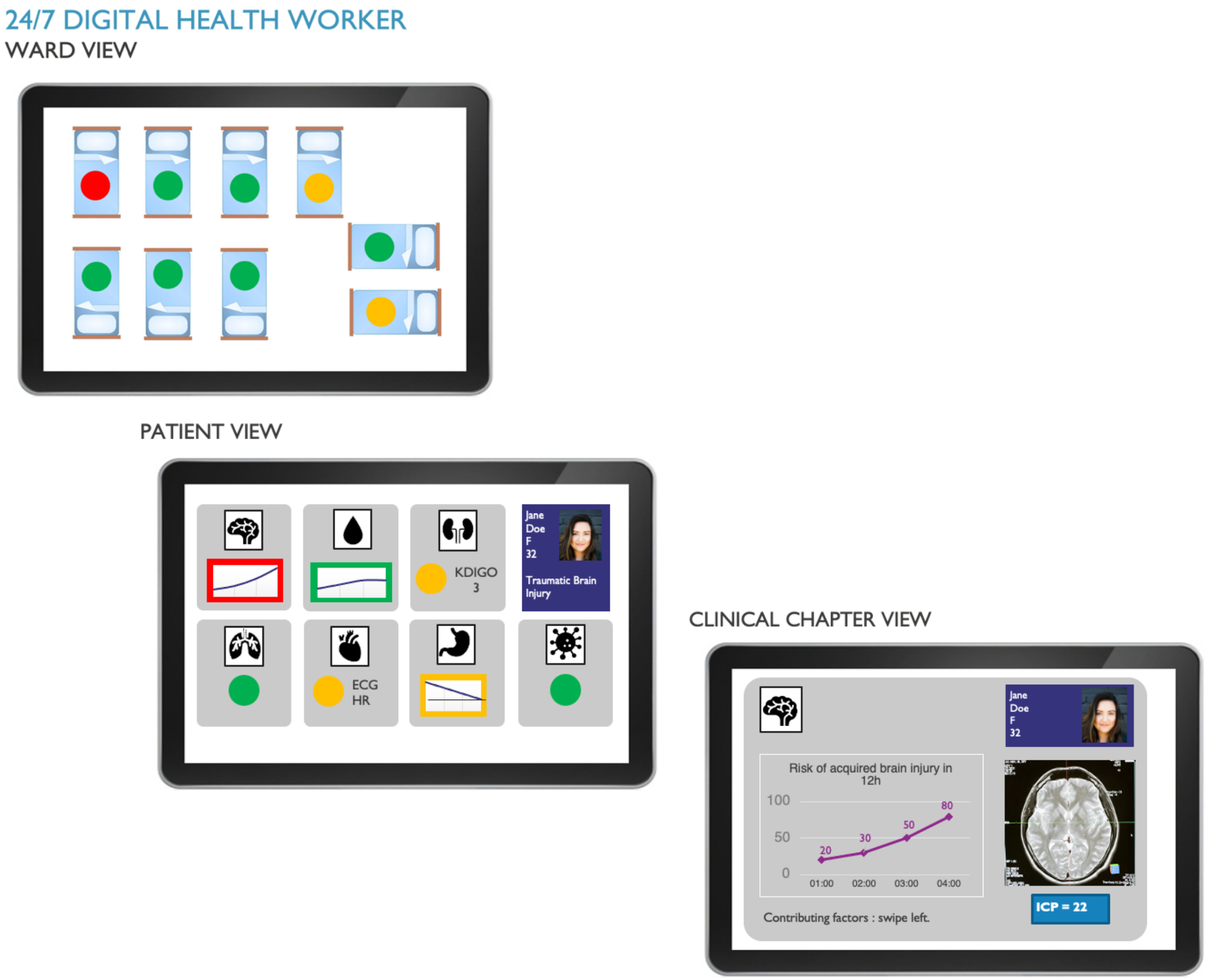

---

[1] By data analytics we mean some computation on monitoring data that allows for a better interpretation of this monitoring data. This can go from very simple to complex:
- indications of min / max values that are exceeded for 1 data stream;

up to and including
- the combination of different data streams and EMR data to display personalized indicators or predictors, e.g. the chance that a patient with a specific medical history will develop brain trauma within 24 hours given brain scans, heart rate, body temperature, respiration and APACHE score.

PART 2*: FOR ALL*

Attitude towards the solution (5')

Q14: What do you think about our proposal?

Q15: Which analytical solution(s) would you prefer to see deployed in the context of this monitoring system?

NUDGE (only if no immediate answer, or to refine the answer given):

1. Smart trends / alarms
2. (Real-time) decision support
3. Clinical pathologies
4. Anomaly detection

Drivers & barriers (5')

Q16: What, who, and which incentive would be needed to bring our solution to your unit? What would you pay for this solution?

Attitude towards sharing / collaboration (10')

Ed.: We think that sharing data and knowledge could be a blocking factor. Hence these next questions.

Q17: Would you contribute patient data to improve data-driven (solutions for) care? If so, under what conditions?

Q18: Would you like to have access to more (internal or external) patient data to improve data-driven (solutions for) care? If so, under what conditions?

Q19: Would you like to share / offer your own data-driven solutions to other healthcare centers to improve their care workflows? If so, under what conditions?

Q20: Would you like to use data-driven software developed in other care centers / by other stakeholders? If so, under what conditions?

Q21: Would you like to collaborate with (other) healthcare centers to develop better data-driven solutions for healthcare? Why / why not?

Q22: Do you think data-driven software developed in-house can improve through collaboration with other stakeholders / care centers?

Closing

Q23: Would you like to stay informed about our innovation project?

Q24: Is there anyone else we should talk to?

Q25: Are there any issues that have not yet been discussed?

(Business cards, next steps)

End of Interview

Practical guidelines for user assumption testing

Overall

Pay attention to:

- Satisficing (compliments): "What do you think about my idea?"
- Steering questions: "What is missing in the current solutions?"
- Fluffy questions and dito replies (anchor to past experiences): "How do you usually carry out that task?"

Further guidelines listed per interview section.

Introduction

- Who are you going to talk to?
- How are you presenting yourself and your project/innovation?
- Do's & Don'ts:
  - Do: introduce yourself & indicate why you interview the person
  - Don't: give away too much information on your innovation
  - Do: capture all relevant information to later on segment and profile your interviewee
  - Don't: start asking difficult or complex questions
  - Do: ask to record the interview
  - Don't: make too extensive notes while interviewing

Current practices

Do's & Don'ts:

- Do: create a time-line / process overview based on the responses & validate it directly

- Don't: steer the conversation too much or pre-answer, be open for unexpected stuff
- Do: ask for clarification when unknown terms are used
- Don't: forget to ask about positive aspects (delights)
- Do: refer to past actions and use open questions – why / how
- Don't: be judgmental or steering

Unmet needs & wants

Do's and don'ts

- Do: let the respondent indicate positive and negative aspects in the process/timeline
- Don't: start by mentioning specific needs yourself
- Do: let the respondent prioritize the needs and wants they elicit
- Don't: keep on asking if there are other needs left
- Do: check whether the need is shared by others
- Don't: forget to ask questions to further specify the needs & wants (dig deeper)

Innovation pitch

Short & to-the-point!!!
Use visuals or tangibles (demo) if possible.
Don't make it too technical, focus on added value without going into sales-mode.

Attitude towards the solution

Do's and don'ts

- Do: let room for spontaneous reactions and opinions, but urge them to be specific
- Don't: filter out negative feedback
- Do: try to reflect back to the 'current state' of the respondent to make it more tangible
- Don't: go in defense-mode
- Do: use card sorting or other techniques for feature prioritization or package creation
- Don't: accept all feature requests, try to understand the 'why' behind them

Drivers & barriers + Value capture

Do's and don'ts

- Do: only do this near the end of the interview
- Don't: steer away from potential barriers or hide them
- Do: use information from the 'current state'
- Don't: use this as the only info for pricing (triangulation)
- Do: try a 'role-taking' exercise
- Don't: push too much if they are not that interested

Closing

Thank the respondent!
Ask if they want to be kept up-to-date and willing to be involved in later stages.
Ask for other interesting interviewees (snowballing).

Supplementary Note 2: Noteworthy models

In this appendix we go into some more detail about the most relevant models that we found.

eICU models: The most interesting repositories that we found were:

- Palepu et al.[1]: Predicting outcome for Traumatic Brain Injury. The authors gain approximately 0.06 AUROC over APACHE IV. Whilst clinically relevant, the model is not reproducible: the authors state the code is only available on request and so is not public, and it is not clear what the licensing conditions would be for any given interested user.
- [2]: AKI prediction in next 6 hours. Clinically relevant, but not reproducible (repository exists, but no license).
- Rocheteau et al.[3] : Length-of-stay prediction. The repository exists and has a usable license. However, not clinically relevant according to the VoC.
- Tang et al.[4]: About a preprocessing pipeline to support modelling with MIMIC and eICU data. Also includes some clinically relevant models as a use-case. Not reproducible: repository exists, but no license.
- Abad et al.[5]: Reproducible, but not clinically relevant. The article is mostly about evaluating the usefulness of a set of features rather than proposing a model as such, and the clinical relevance of the predictive target (discharge destination) is not that good.

In all the above references the entire eICU dataset was used as input, and no specific attempt was made to evaluate models built on a subset of centres and tested on a different subset.

MIMIC-III models: The most interesting other repositories that we found were:

- Lin et al.[6]: Unplanned ICU readmission prediction. The work looked very interesting but is not reproducible: the repository had no license and upon assessing the code there were some questions as to whether it would actually run.
- Kaji et al.[7]: Predict daily sepsis, myocardial infarction (MI), and antibiotic administration. Whilst this work passed all of our requirements, the article concludes that the model is mostly relying on features that are proxies for clinical decision making, and as such we assessed the model to be less than ideal. Nonetheless, we commend the authors for their efforts to make the code public and usable.

- Pakbin et al.[8]: The use-case is readmission prediction. Clinically relevant and reproducible: The repository exists and has a usable license. This is the model we chose; it is further described in the main text.

Supplementary References

[1] Anil K Palepu, Aditya Murali, Jenna L Ballard, Robert Li, Samiksha Ramesh, Hieu Nguyen, Hanbiehn Kim, Sridevi Sarma, Jose I Suarez, and Robert D Stevens. Digital signatures for early traumatic brain injury outcome prediction in the intensive care unit. *Sci. Rep.*, 11(1):19989, October 2021.

[2] Hao Du, Ziyuan Pan, Kee Yuan Ngiam, Fei Wang, Ping Shum, and Mengling Feng. Self-correcting recurrent neural network for acute kidney injury prediction in critical care. *Health Data Science*, 2021:1–10, December 2021.

[3] Emma Rocheteau, Pietro Liò, and Stephanie Hyland. Temporal pointwise convolutional networks for length of stay prediction in the intensive care unit. In *Proceedings of the Conference on Health, Inference, and Learning*, CHIL '21, page 58–68, New York, NY, USA, 2021. Association for Computing Machinery. ISBN 9781450383592. doi: 10.1145/3450439.3451860. URL `https://doi.org/10.1145/3450439.3451860`.

[4] Shengpu Tang, Parmida Davarmanesh, Yanmeng Song, Danai Koutra, Michael W Sjoding, and Jenna Wiens. Democratizing EHR analyses with FIDDLE: a flexible data-driven preprocessing pipeline for structured clinical data. *J. Am. Med. Inform. Assoc.*, 27(12):1921–1934, December 2020.

[5] Zahra Shakeri Hossein Abad, David M Maslove, and Joon Lee. Predicting discharge destination of critically ill patients using machine learning. *IEEE J. Biomed. Health Inform.*, 25(3):827–837, March 2021.

[6] Yu-Wei Lin, Yuqian Zhou, Faraz Faghri, Michael J Shaw, and Roy H Campbell. Analysis and prediction of unplanned intensive care unit readmission using recurrent neural networks with long short-term memory. *PLoS One*, 14(7):e0218942, July 2019.

[7] Deepak A Kaji, John R Zech, Jun S Kim, Samuel K Cho, Neha S Dangayach, Anthony B Costa, and Eric K Oermann. An attention based deep learning model of clinical events in the intensive care unit. *PLoS One*, 14(2):e0211057, February 2019.

[8] A. Pakbin, P. Rafi, N. Hurley, W. Schulz, M. Harlan Krumholz, and J. Bobak Mortazavi. Prediction of icu readmissions using data at patient discharge. In *2018 40th Annual International Conference of the IEEE Engineering in Medicine and Biology Society (EMBC)*, pages 4932–4935, 2018. doi: 10.1109/EMBC.2018.8513181.
